# Sativex (nabiximols) for the treatment of Agitation & Aggression in Alzheimer’s dementia in UK nursing homes: a randomised, double-blind, placebo-controlled feasibility trial

**DOI:** 10.1093/ageing/afaf149

**Published:** 2025-06-06

**Authors:** Christopher P Albertyn, Ta-Wei Guu, Petrina Chu, Byron Creese, Allan Young, Latha Velayudhan, Sagnik Bhattacharyya, Hassan Jafari, Simrat Kaur, Pooja Kandangwa, Ben Carter, Dag Aarsland

**Affiliations:** Centre for Healthy Brain Ageing, Department of Psychological Medicine, King’s College London Institute of Psychiatry, Psychology & Neuroscience, London, UK; Centre for Healthy Brain Ageing, Department of Psychological Medicine, King’s College London Institute of Psychiatry, Psychology & Neuroscience, London, UK; Division of Psychiatry, Department of Internal Medicine, China Medical University Beigang Hospital, Beigang, Taiwan; Department of Biostatistics and Health Informatics, King’s College London Institute of Psychiatry Psychology & Neuroscience, London, UK; Department of Life Sciences, Brunel University London, London, UK; School of Academic Psychiatry, King’s College London Institute of Psychiatry Psychology & Neuroscience, London, UK; Centre for Healthy Brain Ageing, Department of Psychological Medicine, King’s College London Institute of Psychiatry, Psychology & Neuroscience, London, UK; School of Academic Psychiatry, King’s College London Institute of Psychiatry Psychology & Neuroscience, London, UK; Department of Biostatistics and Health Informatics, King’s College London Institute of Psychiatry Psychology & Neuroscience, London, UK; Centre for Healthy Brain Ageing, Department of Psychological Medicine, King’s College London Institute of Psychiatry, Psychology & Neuroscience, London, UK; Centre for Healthy Brain Ageing, Department of Psychological Medicine, King’s College London Institute of Psychiatry, Psychology & Neuroscience, London, UK; Biostatistics and Health Informatics De Crespigny Park Denmark Hill, King’s College London, London SE5 8AF, UK; Division of Academic Psychiatry - Old Age Psychiatry, Institute of Psychiatry Psychology and Neuroscience, London, UK; Centre for Age-Related Medicine, Stavanger University Hospital, Stavanger, Rogaland, Norway

**Keywords:** dementia, Alzheimer’s disease, cannabinoid-based medicine, neuropsychiatric symptoms, behavioural and psychological symptoms of dementia, older people

## Abstract

**Background:**

Alzheimer’s Disease (ad) patients often experience clinically significant agitation, leading to distress, increased healthcare costs and earlier institutionalisation. Current treatments have limited efficacy and significant side effects. Cannabinoid-based therapies, such as the nabiximols oral spray (Sativex®; 1:1 delta-9-tetrahydrocannabinol and cannabidiol), offer potential alternatives. We aimed to explore the feasibility and safety of nabiximols as a potential treatment for agitation in ad.

**Methods:**

The ‘Sativex® for Agitation & Aggression in Alzheimer’s Dementia’ (STAND) trial was a randomised, double-blind, placebo-controlled, feasibility study conducted in UK care homes. Participants with probable ad and predefined clinically significant agitation were randomised to receive placebo or nabiximols for 4 weeks on an up-titrated schedule, followed by a 4-week observation period. To be considered feasible, we prespecified the following thresholds that needed to be met: randomising 60 participants within 12 months, achieving a ≥ 75% follow-up rate at 4 weeks, maintaining ≥80% adherence to allocation and estimating a minimum effect size (Cohen’s d ≥ 0.3) on the Cohen–Mansfield Agitation Inventory. This trial is registered with ISRCTN 7163562.

**Findings:**

Between October 2021 and June 2022, 53 candidates were assessed; 29 met eligibility criteria and were randomised. No participants withdrew, and adherence was high (100%) and was generally feasible to deliver. The intervention was well tolerated (0 adverse reactions), with no safety concerns reported.

**Interpretation:**

Despite significant COVID-19 pandemic related challenges, administering nabiximols through oral mucosa to advanced ad patients with agitation demonstrated feasibility and safety. These findings support a larger confirmatory efficacy trial to evaluate the potential therapeutic efficacy of nabiximols for agitation in ad.

## Key Points

- Cannabinoid oral spray feasibility demonstrated for agitation in Alzheimer’s patients.
- Low-dose mixed delta-9-tetrahydrocannabinol and cannabidiol showed favourable safety profile and high tolerability.
- Future studies should consider dose-finding and inclusion of community-dwelling participants in addition to care home residents.
- Recruitment challenges due to COVID-19 pandemic limited study’s statistical power.
- Successful feasibility and tolerability support exploration of cannabinoid intervention in a larger phase 3 confirmatory trial.

## Introduction

Alzheimer’s dementia (ad), a progressive neurodegenerative disorder, is characterised by a decline in cognitive function, which significantly impacts daily living and quality of life [1]. Behavioural and psychological symptoms of dementia (BPSD)—such as agitation, aggression, delusions, hallucinations, depression and sleep disturbances—are particularly challenging and affect up to 90% of individuals with dementia [2]. Agitation, a common BPSD symptom, involves excessive motor activity and aggression, is associated with increased caregiver burden, healthcare costs and institutionalisation [3, 4].

In the UK, care home residents with dementia frequently exhibit agitation, posing significant challenges to care providers. Approximately 70% of care home residents in the UK have dementia, with a substantial proportion experiencing severe BPSD [5]. The management of agitation in this population is complex, often requiring a combination of pharmacological and nonpharmacological interventions. However, current pharmacological treatments, including most antipsychotics and benzodiazepines, are associated with limited efficacy and significant adverse effects, such as increased risk of falls, sedation and cardiovascular events [6, 7]. Consequently, there is an urgent need for safer and more effective therapeutic options.

Recent advances in cannabinoid psychopharmacology have highlighted the potential of cannabinoid-based therapies in managing BPSD, particularly agitation [8]. Cannabinoids, the active compounds found in the cannabis plant, interact with the endocannabinoid system, which plays a crucial role in regulating mood, cognition and behaviour [9–11]. Nabiximols (Sativex®), an oromucosal spray containing a balanced ratio of delta-9-tetrahydrocannabinol (THC) and cannabidiol (CBD), has emerged as a promising candidate for this purpose. It is currently licensed for the treatment of spasticity in multiple sclerosis, but its potential benefits may extend beyond this indication [12, 13], and its administration route bypassing liver metabolism and gastrointestinal tract might offer more direct effect with less adverse reaction.

In this study, we aimed to explore the feasibility of nabiximols for people living with ad and agitation in care homes.

## Methods

### Study design

The Sativex® for Agitation & Aggression in Alzheimer’s Dementia (STAND) study was a double-blind, parallel-group, randomised placebo-controlled trial assessing the feasibility of nabiximols (Sativex) for treating agitation and aggression in Alzheimer’s dementia. The trial received ethics approval from West Midlands—Coventry & Warwickshire Research Ethics Committee). The protocol, statistical analysis plan (SAP) and study materials are available [14, 15].

### Participants

Participants aged 55–95 years with a probable ad diagnosis, and clinically significant agitation/aggression, were recruited from existing care homes within the greater London area, primarily through the National Institute of Health and Social Care Research (‘NIHR’) Maudsley Biomedical Research Centre Care Home Research Network. Full eligibility criteria can be sourced from the protocol and ISRCTN preregistration. Witnessed informed consent was obtained from the participant or a personal/professional legal representative either in-person or via post or electronic consent.

### Randomisation and masking

At the end of baseline assessments, participants were randomised (1:1) to receive either nabiximols sprays containing 2·7-mg THC/2·5-mg CBD or placebo sprays with the same peppermint oil flavouring/colourings. Randomisation was stratified by baseline ad severity [low: Functional Assessment Staging Tool (FAST) ≤ 5, moderate: FAST = 6, severe: FAST = 7] and sequence was generated using randomly varying block (sizes of two and four) by King’s Clinical Trials Unit (KCTU) and hosted on a web-based system hosted by KCTU. Treatment allocation was blinded to study researchers, participants, family caregivers and care home staff. Care home staff nurses administered sprays according to a standardised dosing schedule.

All parties remained blinded to the treatment allocation until after the datalock and presentation of the study results. The trial statistician was kept blinded until approval of the SAP by the independent Trial Steering Committee (TSC), at which point the trial statistician was able to review data partially blinded (arms labelled A/B) to prepare the analysis code. After database lock, the trial statistician was able to request data fully unblinded to prepare the final report.

### Procedures

The target dose was four sprays/day of nabiximols (10.8-mg THC/10-mg CBD) or placebo, titrated up from one spray per day for the first 3 days to a maximum dose of four sprays/day. The intervention was up-titrated for a total treatment duration of 4 weeks. The participants were then checked 6 and 8 weeks postbaseline (2 and 4 weeks after completing the treatment schedule) for safety and other outcome measures. The researchers either telephoned or visited care homes in-person at baseline and Weeks 2, 4, 6 and 8 and completed outcome assessments concerning safety, adherence and clinical outcomes. The assessments on Weeks 6 and 8 were in particular designed to evaluate any potential lasting response or withdrawal effect. Participants with issues such as side-effects, physical conditions or noncompliance limiting dose-escalation, were checked by the study doctor and the principal investigator to confirm whether they should remain on the current dose or be withdrawn from the trial.

### Outcomes

The primary objective was to assess feasibility, defined by four key criteria: randomising 60 participants within 12 months, achieving a ≥ 75% follow-up rate at 4 weeks, maintaining ≥80% adherence to allocation and estimating a minimum effect size (≥0.3) on the Cohen–Mansfield Agitation Inventory (CMAI) score at Week 4.

Safety and tolerability were assessed through self- and carer-reported side effects, adverse events (AEs) and suicidality (Columbia-Suicide Severity Rating Scale, C-SSRS). Secondary clinical outcomes included CMAI and Neuropsychiatric Inventory-Nursing Home Version (NPI-NH) scores, collected fortnightly from Weeks 0–8 (not including CMAI at Week 4). Additional secondary outcomes—such as Quality of Life (QOL-ad, QUALID), Abbey Pain Scale (APS) and standardised mini-mental state examination (sMMSE)—were collected at baseline and Weeks 4 and 8. FAST of AD and Clinical Frailty Scale (CFS) scores were collected at baseline and Week 4 only. For all assessments (except the sMMSE), a lower score represents an improvement in symptoms/quality of life/functioning.

### Sample size and statistical analysis

A sample size of 60 (1:1) would have enabled study researchers to estimate a drop-out rate of 20% to within a 95% confidence interval (CI) of ±10%. Primary feasibility analyses were conducted on all available data.

The TSC approved the SAP prior to the analyst gaining access to the trial arm (coded A/B). A modified intention to treat analysis was used for clinical outcomes that included randomised participants with a baseline and at least one follow-up measurement of the outcome. Analysis was conducted on Stata version 18.

For the primary feasibility outcomes, the number and relevant proportions of participants randomised, retained and deemed adherent were reported alongside 95% CIs specifying exact binomial distributions. As CMAI may be the potential primary outcome in a future trial, a progression criterion was included in the primary feasibility metrics to provide an initial assessment of potential effect size although with the caveat that this sample would not provide any powered conclusions.

Demographics and secondary outcome measures were summarised using descriptive statistics by arm and overall at each timepoint. The mean difference between arms for secondary outcome measures were estimated using mixed linear models adjusting for the fixed effects of arm, baseline disease severity, baseline measurement of the outcome, time and an arm*time interaction term. Marginal treatment effects were reported for each follow-up timepoint with an adjusted mean difference (aMD). A random intercept was fitted at the participant level to account for repeated measurements. For secondary outcomes only measured at baseline and one follow-up timepoint (i.e. FAST and CFS), no fixed effect for time and arm*time was included and no random intercept was included. For heavily skewed continuous measures, the relevant score categories were used instead in similar logistic regression models. Standardised treatment effect estimates (Cohen’s d) for continuous outcomes were calculated by dividing mean differences between arms by the pooled baseline standard deviation (SD) of the outcome. No significance level was set and no *P*-values were reported as the aim was to provide a range of preliminary effect estimates.

## Results

**Figure 1:**
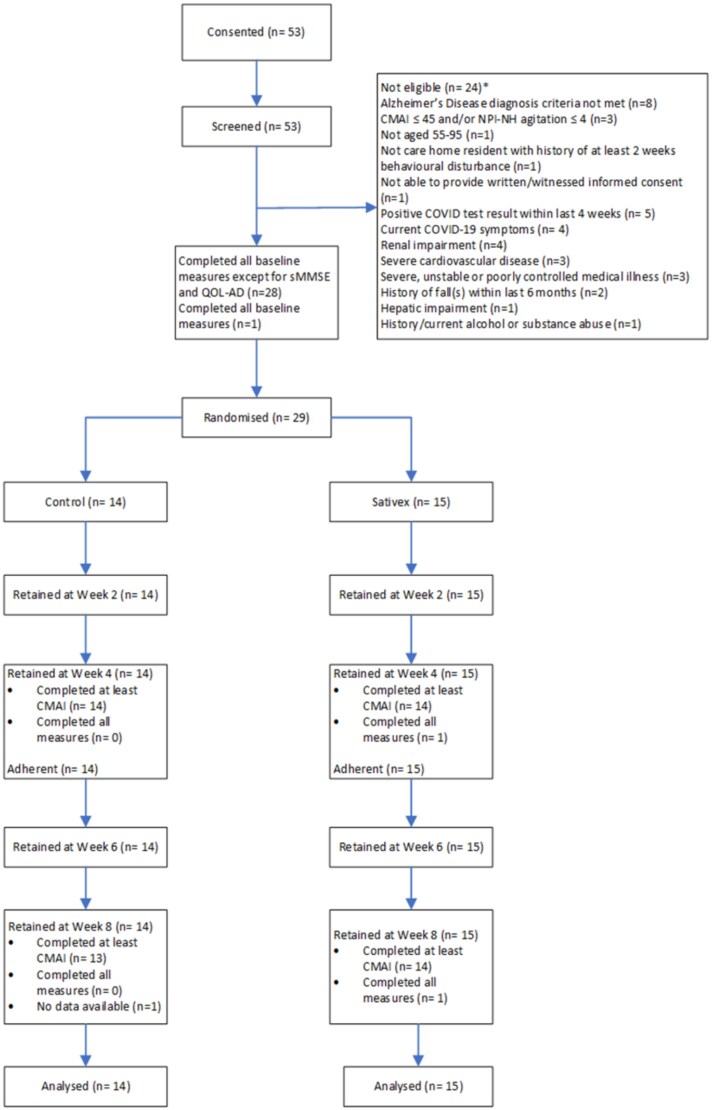
Trial profile. ^*^Screened residents may have been ineligible for more than one reason.

Between 17 November 2021 and 4 July 2022, 53 nursing home residents consented and were assessed for eligibility. The COVID-19 pandemic, particularly the Omicron wave, disrupted recruitment, leading to reduced screening and a pause in trial activities. Ultimately, 29 participants (55% of those assessed) were randomised after excluding 24 ineligible participants (14 to placebo, 15 to nabiximols). All randomised participants completed the 8-week follow-up period and were included in primary analyses (Figure 1).

Table 1 shows baseline demographic and clinical characteristics of the participants. The two groups were similar at baseline in terms of age, sex, severity of cognitive decline and APS, but the nabiximols group had higher baseline agitation severity and overall BPSD scores. A description of current psycholeptic medications (at baseline and continued throughout the trial) between treatment groups is included as a table in the supplementary materials.

**Table 1:** Baseline demographics and clinical characteristics of participants.

Demographics/characteristics	Nabiximols (*n* = 15)	Placebo (*n* = 14)
Age^a^	81·7 (7·9)	80 (8·6)
Sex		
Male	6 (40%)	9 (64%)
Female	9 (60%)	5 (36%)
Disease severity		
Moderate (FAST ≤5)	0 (0%)	0 (0%)
Moderately severe (FAST = 6)	5 (33%)	4 (29%)
Severe (FAST = 7)	10 (67%)	10 (71%)
Mean CMAI total score (SD)	95.5 (29·0)	71.5 (26·3)
Mean NPI-NH total score (SD)	58.5 (24·3)	30·2 (20·9)
Mean NPI-NH disruptiveness score (SD)	16.3 (11·4)	10.2 (8·4)
APS category		
No pain	14 (93%)	12 (86%)
Mild pain	1 (7%)	2 (14%)
Moderate pain	0 (0%)	0 (0%)
Severe pain	0 (0%)	0 (0%)

Data are *n* (%).

^a^The age at randomisation was skewed, and the median (IQR) of the two groups were 83·0 (77·0–86·0) and 81·0 (75·0–87·0), respectively.

The primary feasibility outcome measures are summarised in Table 2. Retention was 100% (29/29) at both Weeks 4 and 8 follow-ups, surpassing the 75% target. Adherence was 100% (29/29); participants received at least one dose per week and generally adhered to the titration schedule (see Supplementary Table 1 for the titration progress and summaries of intervention experiences). However, the clinical effect size for CMAI did not reach the desired threshold (Cohen’s d ≥ 0·3), showing 0·23 at Week 4 and 0·08 at Week 8.

**Table 2:** STAND primary feasibility progression outcome measures.

Primary feasibility metric and parameter	Estimate
To consent and randomise 60 participants as measured by number of participants randomised at trial close	Twenty-nine (29) randomised by trial close
To follow up at least 75% of those randomised as measured by the number of participants completing	29/29 retained at Week 4
each follow up to and including the secondary endpoint (Week 8)	29/29 retained at Week 8
	Retention: 100% (95% CI: 88%)
For a minimum of 80% of participants to demonstrate adherence to the allocation. Adherence defined by	29/29 had at least 1 dose/week.
participants receiving at least one dose per week during treatment phase (Weeks 1–4)	Adherence: 100% (95% CI: 88%)
To estimate an effect size of at least 0·3 for CMAI (Using Cohen’s d)	Adjusted analyses (control as reference group)
	Week 4: 0·23 (95% CI: −0·2 to 0·7)
	Week 8: 0·08 (95% CI: −0·4 to 0·5)

One-sided 97·5% CI given if proportion is 100%. About half of participants screened were found eligible and subsequently randomised. From the first randomisation on 17 November 2021 to the last randomisation on 4 July 2022, this represents 29 randomisations over a recruitment period of 7.5 months. On average, 3·9 participants were randomised per month (95% CI, 2·6–5·6).

In this trial, 21% (6 of 29) of participants reported at least one nonserious AE; all were mild severity and unrelated to the study intervention. The AE listing included hepatobiliary, respiratory, eye and skin/tissue disorders, with mild cases across the placebo (*n* = 2) and nabiximols (*n* = 4) arms. Serious adverse events (SAEs) occurred in two participants (one in nabiximols arm and one participant in placebo arm with two events), with moderate or severe respiratory and renal/urinary disorders. No AEs nor SAEs were attributed to the intervention, and there were no reported deaths, falls, increase in C-SSRS or withdrawals (see Supplementary Table 2 for summary of AEs and SAEs by trial arm).

**Figure 2:**
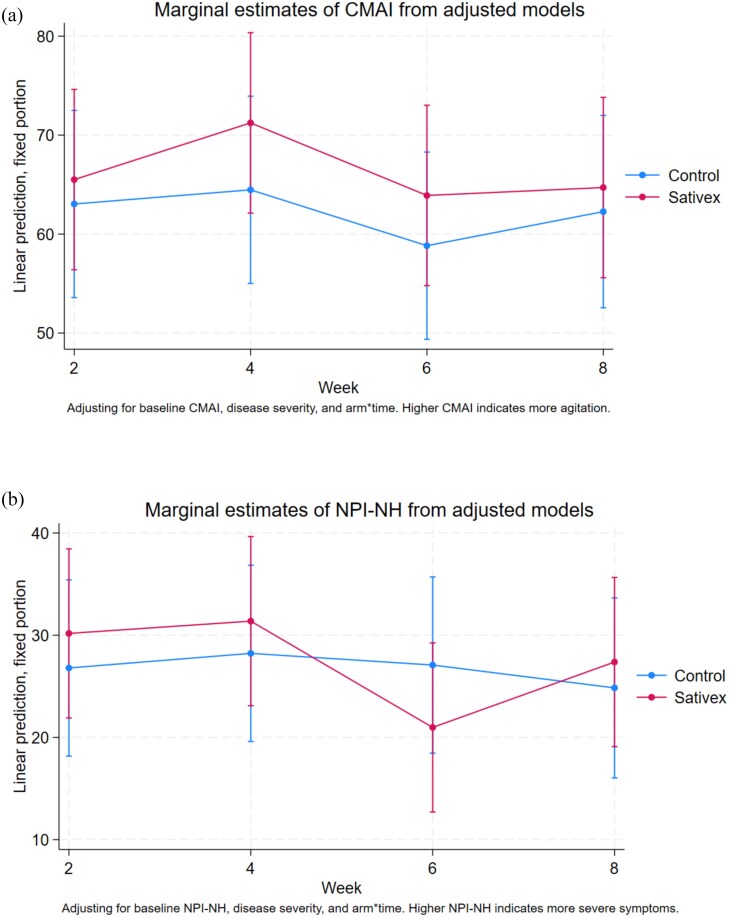
Marginal estimate for CMAI (a) and NPI-NH (b) scores by treatment arm.

The results of the adjusted analysis for secondary clinical and neuropsychiatric outcomes are shown in Table 3, and the full secondary neuropsychiatric outcome data by trial arm and timepoint can be found in Supplementary Table 3 and supplementary figures. At the end of the treatment period by Week 4, CMAI scores had improved in both groups, with mean scores reducing to 58·2 (SD 23·9) in the control and 77·0 (SD 24·5) in the nabiximols group. There was no association between nabiximols and total CMAI at Week 4 with an aMD of 6·77 (95% CI −6·71 to 20·25; Cohen’s d = 0·23) or 4 weeks post-treatment at Week 8. Similar trends were observed in NPI-NH scores, which decreased in both groups at Week 4 and further by Week 8, as shown in Figure 2. QUALID and FAST scores showed no significant changes between groups, and the adjusted effect sizes did not reach clinically meaningful thresholds. By Week 8, mean adjusted differences between nabiximols and control groups for CMAI, NPI-NH and QUALID remained minimal (CMAI: 2·43; NPI-NH: 2·53; QUALID: −2·61).

**Table 3:** Results of adjusted analyses for secondary clinical and neuropsychiatric outcomes.

Outcome	N in analysis model	Mean difference (95% CI)	Cohen *d* (95% CI)
CMAI	29		
4 weeks		6·77 (−6·71, 20·25)	0·23 (−0·23, 0·68)
8 weeks		2·43 (−11·23, 16·10)	0·08 (−0·38, 0·54)
NPI-NH	29		
4 weeks		3·15 (−9·46, 15·76)	0.12 (−0·36, 0·59)
8 weeks		2·53 (−10·2, 15·26)	0·10 (−0·38, 0·57)
QOL-ad	0		
4 weeks		N/A	N/A
8 weeks		N/A	N/A
QUALID2	29		
4 weeks		−0·25 (−4·21, 3·7)	−0·03 (−0·47, 0·42)
8 weeks		−2·61 (−6·63, 1·41)	−0·29 (−0·75, 0·16)
APS^a^	29		
4 weeks		1·08 (0)^b^	N/A
8 weeks		3·82 (0·28, 52·09)^b^	N/A
sMMSE			
4 weeks		N/A	N/A
8 weeks		N/A	N/A
FAST at 4 weeks	29	−1·22 (−2·23, −0·21)	-0·71 (−1·30, −0·12)
CFS at 4 weeks^a^	19	1·13 (0·06, 21·09)^c^	N/A

^a^Odds ratios (ORs) reported instead of mean differences for APS and CFS measures.

^b^All participants had reported APS scores in the ‘No pain’ category at Week 4; there were no values in the ‘Mild pain’ cells. There were only four participants reporting APS scores in the ‘Mild pain’ category at Week 8.

^c^Only two participants reported CFS scores in either the ‘Living with very mild frailty’ or ‘Mild frailty’ categories at Week 4 and some observations were omitted because they perfectly predicted being in the combined lower categories.

Pain levels, as measured by the APS, remained low across groups, with no moderate or severe pain reported throughout the study. A small decrease (improvement) in dementia function staging was observed at Week 4 (aMD: −1·22 [−2·23, −0·21]) but only two participants in the nabiximols arm actually improved enough at Week 4 to move from the ‘severe’ disease category to the ‘moderately severe’ disease category.

## Discussion

For two of the prespecified primary feasibility thresholds the STAND trial demonstrated the safety and feasibility of conducting a randomised, placebo-controlled study of cannabinoid oral spray for agitation in patients with AD. Despite under-recruitment, most screened patients participated, with high retention and adherence throughout the trial (to the treatment and for completing outcome measures). No AEs attributable to the intervention were identified, and the safety profile appeared to be similar between the nabiximols arm and the placebo arm. Unfortunately, it is not possible to determine whether the target recruitment would have been possible due to pandemic related recruitment difficulties, and therefore, the estimated effect size calculation was also compromised. A future study should incorporate interim analyses to assess these criteria.

The recruitment challenges should be considered in the context of recruiting in care homes during the COVID pandemic. Critically, the initial planned 12-month recruitment window was reduced to 7.5-months, resulting in 4.5 fewer months than originally planned to reach the recruitment target of 60 participants (see Table 2 for precise dates of first and last randomisations). Even within these 7.5-months, recruitment had been paused because of the Omicron wave in the earlier months, and for ~6-weeks to resupply our stock of drug and placebo (which had expired since first receiving in early 2020 when the trial was initially planned to start). Further, the active recruitment window could be argued to be closer to ~6 months. Accounting for only having potentially ~50% of the planned recruitment window, intermittent trial pauses, and with our recruitment rate accelerating in the later months, we cautiously argue that it may be that the sample target would have been reached if the trial was operational for the full planned 12 months. The unprecedented burnout among care home staff globally limited the capacity for clinical research [16], even as residents likely experienced escalating BPSD [17]. Additionally, the pandemic contributed to attrition through increased mortality, participant ineligibility due to presence of covid symptoms and care home closures across the UK—all of which impacted the recruitment and randomisation of the study. Despite challenges, retention reached 100% and data completion for key neuropsychiatric outcomes was robust, supporting the feasibility of administering this intervention.

With regard to the safety profile, although all the participants in this study were classified as either moderately severe or severe dementia, and the majority of the participants (100% in placebo arm and 73% in nabiximols arm) had moderate to severe frailty, there were very few reports of AEs. A similar study conducted by our team, applying pure CBD of a much higher dosage (up to 600 mg daily) in a modest sample, found that 12.5% of participants in the CBD arm had treatment-related dizziness [18]. Another small-sized study done in Australia using higher dosage THC/CBD mixture (up to 50-mg THC and 34-mg CBD daily) also found a significantly higher rate of overall AEs in their treatment arm [19]. Compared to these studies, ours applying a mixture of low dose CBD and medium dose THC appeared to be safer and better tolerated, even in a cohort of frail, late-stage ad patients.

Safety remains the foremost concern in designing interventions for BPSD. Antipsychotics, benzodiazepines and antidepressants are commonly used empirically for these symptoms. However, prior to the approval of brexpiprazole—a partial agonist at dopamine D2 and D3 receptors—for agitation in AD, no medication was considered safe for long-term use [20]. Even brexpiprazole carries a black box warning, highlighting the increased risk of mortality associated with antipsychotic use in older adult patients with dementia-related psychosis [7]. Benzodiazepines are known for their negative impact on the cognition and postural balance of older adults [21]. Antidepressants like citalopram and mirtazapine have limitations too: citalopram may reduce agitation but can impair cognition and prolong QT interval [22], while mirtazapine showed no benefit for agitation in a large RCT and might increase mortality risk [23]. Given the favourable safety and tolerability profile shown at the current dose, we would recommend a further dose-finding study to establish the minimal effective dose before initiating a larger phase-III trial. The STAND trial focused on a nursing home population, allowing for continuous safety and compliance monitoring. However, based on our positive safety results, future studies should consider including community-dwelling participants to enhance recruitment and reach a broader patient population in need.

While the adjusted analyses indicated little evidence of a signal from nabiximols, this study was not powered to demonstrate a clinical effect. The small sample size and lack of precision should be considered when interpreting the effectiveness of the intervention, along with the finding that only half of participants completed the full titration schedule as planned. In addition, agitation is likely a heterogeneous and multifactorial syndrome that needs to be defined and measured with more specificity, as recommended previously by the SYMBAD study [23]. Although using the questionnaires such as the NPI and the CMAI for agitation assessment have been widely validated and implemented, wearable device has been proposed to complement these traditional instruments [24, 25]. These device-based measurements might have the potential to reveal information associated with the outcome not easily captured by the questionnaires and therefore stratify the patients and outcomes more specifically. For example, the International Psychogeriatric Association recently suggested the recognition and separation of nocturnal/circadian agitation [3]; wearable devices capable of continuous behavioural measurement might have the potential to distinguish symptoms happen in daytime and at night and therefore facilitate the outcome measurement.

This trial introduces two key innovations relevant to the feasibility, safety and efficacy to be considered for future studies: the use of an oromucosal spray for drug delivery and the application of cannabinoids in dementia care in a dementia cohort with predefined nonpain-related agitation.

It has been noted in a target product profile developed by a consortium of experts that an ideal therapeutic for agitation in ad would address difficulty swallowing (dysphagia) and refusal to swallow [26]. Administering cannabinoid-based medicine (CBM) in dementia patients through oral mucosae bypassing the first-pass effect of liver and the gastrointestinal tract would theoretically result in higher bioavailability, more direct neuropsychiatric response and less adverse effects from the gastrointestinal tract (particularly associated with THC), in addition to preventing swallowing-related issues. To our knowledge, only five RCTs have been published previously examining the effect of cannabinoids on BPSD [8, 19], and ours is the first one testing the oromucosal administration of medication. Addressing common challenges like dysphagia and refusal to swallow, and bypassing the gastrointestinal tract likely contributed to the high tolerability and adherence observed, as well as the very low adverse effect recorded, despite a moderately high dose of THC used in our study.

In addition, our study design recruiting patients with predefined clinical agitation with little or no pain symptom, was crafted to make more precise inference on agitation. However, it potentially nullified the effect, as previous studies show that agitation associated with unpleasant sensory input such as pain might be more responsive to CBM [8]. Future studies might compare nabiximols in agitated dementia patients with and without pain to understand its true effect. Nabiximol might also be considered as an adjunct to sensory interventions—such as music, light and aroma therapy [27, 28]—leveraging CBM’s potential to enhance sensory experiences and regulate pain [11, 12] and circadian rhythm [29, 30]. This combined CBM-sensory approach could enable gradual deprescription, supporting long-term, nonpharmacological management of agitation in ad.

The primary limitations of this study include under-recruitment due to the COVID-19 pandemic, which reduced the statistical power needed to determine effect size, under-dosing, racially homogenous sample, a failure of stratified block randomisation to ensure equal baseline severity of agitation between groups, inadequately tracking rescue medication administration and an over-reliance on proxy-informant measures as a result of pandemic restrictions. Although our sample was slightly larger than those in comparable studies and featured a more balanced sex distribution, the participant numbers remain insufficient to robustly estimate the intervention’s effect size. Additionally, while the relatively low doses of THC and CBD likely contributed to fewer AEs compared with prior studies, these dosages may also be less effective in reducing agitation and aggressive behaviours. The limited sample size warrants cautious interpretation of the safety and efficacy profile in the nabiximols group. The ethnic homogeneity of our sample may limit the generalisability of these findings beyond the UK and Ireland, particularly given the high proportion of white British participants. Also, as our study had significant between-group differences in agitation severity at baseline, a future study should consider a more precise stratification variable other than the FAST to balance baseline agitation severity between groups. Additionally, while we recorded the medications that participants were on at the start the trial, and communicated with the care home that participants must remain on the same dose of their antidepressants, antipsychotics, anti-epileptics, benzodiazepine, lithium and hypnotics dosage, we did not adequately monitor whether rescue medications were administered ‘as needed’ in response to acute episodes of agitation or aggression during the trial period, and thus we do not know if the two groups differed in the administration of ‘as needed’ medications during the study. This level of data should be included for future studies trying to estimate the true effect of the medicinal compound. Finally, an over-reliance on proxy measures meant that we did not collect adequate direct participant clinical information. This is particularly prominent for the sMMSE, a cognitive assessment that we included at the outset as a precautionary exploratory measure, just in case there was an effect (positive or negative) on cognition. Due to pandemic restrictions, it was extremely difficult to complete face–face measures, rendering it uninformative.

This study has three main strengths: a robust design, a favourable safety profile and a high retention rate. Strict inclusion and exclusion criteria, while reducing participant numbers, safeguarded this vulnerable population from SAEs and minimised infection risk among participants, caregivers and researchers. The inclusion of participants with no or minimal pain symptoms also enhances our understanding of the true effect of CBM on agitation in dementia patients. The observed safety profile in this frail, late-stage dementia cohort likely contributed to the 100% retention rate, underscoring the intervention’s feasibility even amid pandemic-related pressures on care home staff. Although, it should be noted that the threshold of at least one dose per week should be updated to be more stringent (perhaps one dose per day).

In conclusion, this study demonstrates the feasibility of a pilot randomised, placebo-controlled trial of nabiximols oral spray for agitation in AD patients in care homes, with no safety concerns observed. The study addresses a novel gap in the literature and clinical practice in the treatment of agitation in AD as (i) it was the first to investigate the social acceptability and tolerability of CBM within care homes in the UK, providing valuable insights into practical administration aspects; (ii) it was the first to evaluate an oral spray formulation of a CBM containing a moderately high dose of THC and a low-dose CBD specifically for dementia patients; and (iii) the clearly predefined. The findings provide insights that can inform future trials in refining dosing schedules and minimising AE risks. However, establishing the intervention’s effect size will require further investigation to assess therapeutic efficacy more definitively. We recommend a larger confirmatory trial be undertaken, incorporating insights from this feasibility study (such as ascertaining a maximum-tolerated dose in this specific patient population), to evaluate nabiximols therapeutic efficacy to treat agitation in AD.

## Supplementary Material

aa-24-2829-File002_afaf149

## Data Availability

A de-identified version of the dataset for meta-analyses or analyses will be available with investigator support from 1 year after the publication via dag.aarsland@kcl.ac.uk. Written proposals will be assessed by members of the King’s Clinical Trials Unit committee, and a decision about the appropriateness of the use of data will be made. A data sharing agreement would need to be put in place before any data is shared.
